# Environmental Risk Factors and Amyotrophic Lateral Sclerosis (ALS): A Case-Control Study of ALS in Michigan

**DOI:** 10.1371/journal.pone.0101186

**Published:** 2014-06-30

**Authors:** Yu Yu, Feng-Chiao Su, Brian C. Callaghan, Stephen A. Goutman, Stuart A. Batterman, Eva L. Feldman

**Affiliations:** 1 Environmental Health Sciences, University of Michigan, Ann Arbor, Michigan, United States of America; 2 Department of Neurology, University of Michigan, Ann Arbor, Michigan, United States of America; University of Florida, United States of America

## Abstract

An interim report of a case-control study was conducted to explore the role of environmental factors in the development of amyotrophic lateral sclerosis (ALS). Sixty-six cases and 66 age- and gender-matched controls were recruited. Detailed information regarding residence history, occupational history, smoking, physical activity, and other factors was obtained using questionnaires. The association of ALS with potential risk factors, including smoking, physical activity and chemical exposure, was investigated using conditional logistic regression models. As compared to controls, a greater number of our randomly selected ALS patients reported exposure to fertilizers to treat private yards and gardens and occupational exposure to pesticides in the last 30 years than our randomly selected control cases. Smoking, occupational exposures to metals, dust/fibers/fumes/gas and radiation, and physical activity were not associated with ALS when comparing the randomly selected ALS patients to the control subjects. To further explore and confirm results, exposures over several time frames, including 0–10 and 10–30 years earlier, were considered, and analyses were stratified by age and gender. Pesticide and fertilizer exposure were both significantly associated with ALS in the randomly selected ALS patients. While study results need to be interpreted cautiously given the small sample size and the lack of direct exposure measures, these results suggest that environmental and particularly residential exposure factors warrant close attention in studies examining risk factors of ALS.

## Introduction

Amyotrophic lateral sclerosis (ALS) is a progressive neurodegenerative disorder involving primarily upper and lower motor neurons in the cerebral cortex, brainstem, and spinal cord [1]. ALS patients often experience difficulty talking, swallowing, breathing and walking, and often develop respiratory insufficiency which is the main cause of death. The prevalence of ALS is 2–9 per 100,000 persons overall and increases with age [2]–[6], and the incidence rate is 1–3 per 100,000 person-years [3], [4], [7]. Although approximately 5–10% of ALS cases can be attributed to genetic factors, the underlying cause of ALS remains largely unknown [8].

A number of epidemiologic studies have suggested that ALS patients have been exposed to environmental toxins [9]–[13]. Environmental risk factors investigated have included, among others, exposures to agriculture chemicals, heavy metals, solvents, electrical magnetic fields (EMF), and exercise [9], [12], [14]–[16]. Results have varied widely in the 40+ epidemiologic studies that have investigated such factors, and an understanding of causal links to environmental agents remains elusive.

The objective of this paper is to evaluate potential environmental risk factors for ALS using a case-control study conducted in the State of Michigan. Additional objectives include describing and evaluating the validity and efficiency of the survey instruments, and discussing key data and methodological issues. Elements of this study form portions of an ongoing study designed to account for interactions among covariates and to utilize biomarkers and other techniques to extend the exposure assessment.

## Materials and Methods

### Recruitment and Case Ascertainment

ALS subjects were recruited through the University of Michigan ALS Clinic with the following inclusion criteria: (1) age greater than 18 years, (2) possible, probable, probable lab-supported, or definite ALS by the revised El Escorial criteria [17], (3) able to provide informed consent, and (4) able to understand and communicate in English. Age- and gender-matched controls were recruited through postings and a University of Michigan website (umclinicaltrials.org) that described the study and eligibility criteria, which included: (1) no diagnosis of ALS or other neurodegenerative condition, (2) no family history of ALS in a first or second degree blood relative, (3) ability to understand and communicate in verbal and written English, and (4) no underlying health condition or medication use that made participation risky, as determined by the study team. Age was matched by decade and gender (e.g., males ages 50–59, females ages 50–59). The study was approved by the Institutional Review Board (Protocol # HUM00028826) at the University of Michigan. All participants provided written informed consent.

### Participant Survey

Participants completed a detailed self-administered written questionnaire that encompassed occupational and residential exposures, residence location, exercise and sports, body weight, tobacco use, military experience, and family history. Questionnaires were mailed to subjects and telephone follow-up was conducted for clarifications, if needed. For patients who had difficulty communicating, the next of kin completed the instrument. Typically, about 90 minutes was needed to complete the fairly lengthy (200 questions, 28 page) questionnaire, which was divided into several sections and used a structured query approach, allowing participants to skip irrelevant sections. Portions of this questionnaire were adapted from those used in the National Health and Nutrition Examination Survey Questionnaire [18].

After requesting standard demographic information, residential history was collected using 54 questions for the current dwelling and 18 questions for each of the three previous dwellings. Questions addressed dates of building construction and occupancy, building type (including detached single family, duplex, multi-family/apartment, mobile home or trailer, other, or unknown) and features (including building materials, floor coverings, presence of basement, presence of outdoor storage, and presence of garage), weatherization, storage of chemicals (e.g., pesticides, solvents, gasoline, and paint among others), and drinking water source (e.g., well or city water). A detailed smoking history was collected, including whether subjects ever smoked and, if applicable, the year smoking started, stopped, the type of tobacco used, and the frequency of smoking. Participants were asked about hobbies, particularly those that might include chemical exposure, such as wood working, metal working, home remodeling, lawn care, automobile repair, small engine repair, and painting. Participants were asked about their physical activity, including the frequency of ten categories of activities (jogging/running, bicycling, swimming, aerobic dancing, recreational dancing, calisthenics, gardening or yard work, weightlifting, playing soccer/football/baseball/field hockey/golf, playing ice hockey/tennis/boxing/wresting) using a five year recall period. In addition, we asked about other activities not mentioned above. Summary or composite measures of physical activity were developed and classified as low, medium, and high based on the number of activities by an individual (defined as 0–3, 4–6, and 7+ activities, respectively).

A detailed occupational history was also requested, and the questionnaire included 24 questions for each of four previous jobs; specifically, their current or most recent job, the previous job, and the two other jobs that were held for the longest period of time. Requested information included job title, industry type, and dates of employment. In addition, subjects were asked to identify occupational exposures, using lists of potential workplace hazards and exposures (e.g., specific chemicals, particles, radiation), the use and presence of personal protective equipment, and hygiene habits (e.g., hand washing).

### Data analysis

Following data entry and consolidation, initial analyses included data cleaning and verification. Dubious or missing entries were checked by either reviewing the original questionnaire, telephone follow up, or as a last resort and if practical, imputed manually based on related questions. Next, using the residence and occupational histories, residence and employment durations were calculated and compared as a test of internal validity. If the residence or job information was inconsistent, such as residing in a different state compared to the workplace, then respondents were queried by telephone and the information revised as indicated. If this attempt failed, then the questioned information was set as missing. Job titles were coded using the Dictionary of Occupational Titles, and workplace types were coded using the North American Industry Classification System. Several new composite variables for specific risk factors were also created; for example, a binary (yes/no) variable called “occupational exposure to heavy metals” combined any positive response to separate questions concerning workplace exposure to arsenic, beryllium, cadmium, chromate, lead, mercury, nickel, and welding fumes. Such variables were created for each job, and also across all jobs. Similarly, composite variables were created for risk factors pertaining to residences and hobbies.

The four exposure time frames that were considered were: (1) no exposure (in the past 30 years), (2) exposure in the last 10 years only (not earlier), (3) exposure 10 to 30 years ago and not in the past 10 years, and (4) continuous exposure over the past 30 years. These time frames were referenced to survey completion in the control population and ALS symptom onset for the case population.

Questions not answered by subjects were interpreted as missing. To obtain a full dataset needed in the conditional logistic regression (CLR) models (described below), missing values were imputed with five replacements. The consistency of imputed data was confirmed with manual checks (for example, the imputed number of years of smoking should be smaller than the subject's age). Subsequent sensitivity analyses confirmed that results obtained using original and imputed data sets were similar.

The survey data were used to generate approximately 100 variables as potential risk factors for each of the four exposure time windows, and a subset of variables for further analysis was selected for use in the CLR models. For continuous variables, univariate statistics (range, mean, medium, quartiles) were calculated and differences between cases and controls were tested using Student t-tests (normally distributed variables) and Kruscal-Wallis tests (non-normal distributions). For categorical variables, cross-tabulations and chi-square tests were used to detect differences between cases and controls. Fisher's exact test was used if the expected count was less than five. Variables showing variability and significant differences were retained for further analyses, as were several variables identified in prior research, such as smoking, physical activity, exposures to metals, and exposures to radiation. Related exposure variables that were moderately to highly correlated (r>0.5) were consolidated as a new variable (e.g., occupational exposures to radiation, x-rays, and electromagnetic fields were grouped into a radiation variable). Stepwise regression was then used to generate final models. A sensitivity analysis for variable entry (with p-values from 0.1 to 0.3) and removal (p-values from 0.15 to 0.35 for variables) showed that in most cases the same variables were selected. Then, odds ratios (OR) and p-values (significance level: p-value <0.1) for potential risk factors were estimated using CLR models for case/control pairs matched on age and gender. Each model included covariates to control for demographics, smoking (cigarette packs per day), physical activity status (low, medium, and high physical intensity groups), and educational attainment. Four models with different exposure conditions (model 1: exposure in the last 30 years, model 2: exposure in the last 10 years, model 3: exposure in the period from 30 years ago to 10 years ago, model 4: continuous exposure in the last 30 years) were constructed to test potential risk factors such that 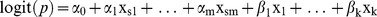
(1)where α =  effect of the stratum (the matching variables age and gender), m =  number of matched sets (strata), β =  log-odds ratio of interested risk factors, x =  risk factor, and k =  number of variables. Several interaction effects were tested (e.g., two-way interactions between education level and exposure risk factors). Because interaction effects were not statistically significant, they were not retained in the final models. Several sensitivity analyses were used to investigate the robustness of the model results to the exposure window.

Data was entered and managed using RedCap [19]. Some analyses used Excel (Microsoft, Redmond, WA, USA). Multiple imputation IVEware 2.0 (Survey Research Center, Institute for Social Research, University of Michigan, Ann Arbor, MI, USA) was used to replace missing data. Statistical analyses were performed using SAS 9.3 (SAS Institute Inc., Cary, NC, USA). The following data represent an interim analysis of an ongoing larger study.

## Results

### Participant Characteristics

The study population was 31% female and averaged (± SD) 61.6±9.0 years of age. Cases and controls had the same age and gender distribution (Table 1), reflecting successful matching. Controls had a higher level of education attainment than cases (p<0.001), with 67% of cases and 95% of controls receiving education beyond high school. Controls were also less likely to be married (p = 0.008, Table 2). When stratified by gender, differences in education were maintained for both males and females, but differences in marital status were found only among males (Table S1). Cases and controls had similar smoking patterns: 55–56% were current or past smokers, 15–18% smoked less than 1 pack per day, and 38–39% smoked greater than1 pack per day (Table 2). Cases had a slightly, but not statistically, longer duration of smoking than controls (mean (SD) years of smoking: 15.7±18.4 for cases and 12.0±14.5 for controls, p = 0.20). Similarly, cases had higher cigarette pack-years (mean  = 20.5±33.6) than controls (mean  = 13.9±20.4), but again, the difference was not statistically significant (p = 0.18). The average cigarette packs per day for cases (0.64±0.88) and controls (0.61±0.69) was similar (p = 0.85). When stratified by gender, smoking status, cigarette packs per day, and the number of years of smoking did not differ between cases and controls; however, female cases had more cigarette pack-years than female controls, due to both slightly higher numbers of heavy smokers (>1 pack/day) and a longer duration of smoking (Table S1). No significant differences were seen for other measures of smoking, including 25th and 75th percentile values of ever-smokers and cigarette packs per day, years of smoking, and cigarette pack-years.

**Table 1:** Demographic characteristics of cases and controls.

Demographics		Cases (n = 66)		Controls(n = 66)		p-value
Variable	Group	Frequency	%	Frequency	%	
Age of consent	40–49	8	12.1	8	12.1	1.000
	50–59	17	25.8	17	25.8	
	60–69	27	40.9	27	40.9	
	70–79	14	21.2	14	21.2	
	80–89	0	0.0	0	0.0	
Gender	Female	31	47.0	31	47.0	1.000
	Male	35	53.0	35	53.0	
Education	≤ High school	22	33.3	3	4.6	<0.001
	> High school	44	66.7	63	95.5	
Marital status	Married*	45	68.2	30	45.5	0.044
	Widowed	7	10.6	5	7.6	
	Divorced	8	12.1	16	24.2	
	Separated	1	1.5	1	1.5	
	Never married	3	4.6	12	18.2	
	Living with partner	2	3.0	2	3.0	

*, significant difference between married and non-married (p = 0.008).

doi:10.1371/journal.pone.0101186.t001

**Table 2:** Smoking and physical activity status by case type.

Variable	Group	Cases (n = 66)		Controls (n = 66)		p-value
		Number	%	Number	%	
Smoker	Never smoker	30	45.5	29	43.9	0.861
	Ever smoker	36	54.6	37	56.1	
Smoking status	Never smoker	30	45.5	29	43.9	0.929
	Former smoker	27	40.9	29	43.9	
	Current smoker	9	13.6	8	12.1	
Cigarette packs/day	Never	30	45.5	29	43.9	0.897
	<1 pack	10	15.2	12	18.2	
	≥1 pack	26	39.4	25	37.9	
Number of years of smoking	Never	30	45.5	29	43.9	0.395
	<20 years	11	16.7	17	25.8	
	≥20 years	25	37.9	20	30.3	
Cigarette pack-years	Never	30	45.5	29	43.9	0.416
	<20 pack-years	13	19.7	19	28.8	
	≥20 pack-years	23	34.8	18	27.3	
Physical activities	Jogging, running	18	27.3	15	22.7	0.547
	Bicycling	33	50.0	33	50.0	1.000
	Swimming	17	25.8	17	25.8	1.000
	Aerobic dancing	10	15.2	9	13.6	1.000
	Recreational dancing	17	25.8	8	12.1	0.029
	Calisthenics	24	36.4	27	40.9	0.592
	Gardening, yard work	54	81.8	46	69.7	0.104
	Weightlifting	17	25.8	18	27.3	0.844
	Soccer, football, baseball, field hockey, golf	24	36.4	17	25.8	0.188
	Ice hockey, tennis, boxing, wresting	9	13.6	5	7.6	0.258
	Other*	15	22.7	16	24.2	1.000
Physical activity intensity (excluding others)	Never	5	7.6	5	7.6	0.245
	Low (0–3 activities)	36	54.5	36	54.6	
	Medium (4–6 activities)	19	28.8	24	36.4	
	Highs (7+ activities)	6	9.1	1	1.5	
Physical activity intensity (including others)	Never	5	7.6	4	6.1	0.090
	Low (0–3 activities)	32	48.5	32	48.5	
	Medium (4–6 activities)	18	27.3	27	40.9	
	Highs (7+ activities)	11	16.7	3	4.6	

*, other activities include ski, fishing, hunting, bowling, yoga, etc.

doi:10.1371/journal.pone.0101186.t002

Involvement in individual sports and other physical activities (Table 2) did not differ between cases and controls, with the exception of recreational dancing (p = 0.029). Similarly, no differences were seen for the number of activities engaged in by participants. Among males, cases were more likely than controls to engage in gardening (p = 0.023) and also in a high intensity of activities when other sports were included (p = 0.054). Among females, the only difference seen was that cases were less likely to dance recreationally (p = 0.038; Table S1). Swimming showed marginally significant differences, but in different directions, when stratified by gender.

### Als Risk Factors

Results of the final CLR models for the entire sample (matched by age and gender) are given in Table 3. Multiple regression model results, stratified by age (adjusted by gender) and gender (adjusted by age), are in Table S2 and Table S3, respectively. Each table includes models for four exposure windows, and each model includes education, smoking, activity, and environmental variables.

**Table 3:** Results of multiple conditional regression models at four exposure windows for all participants.

Risk Factors	1. Exposure in the last 30 years	2. Exposure in the last 10 years	3. Exposure in the period from 30 years ago to 10 years ago	4. Continuous Exposure in the last 30 years
	OR (95% CI)	OR (95% CI)	OR (95% CI)	OR (95% CI)
Education ≥ high school	0.05 (0.01–0.36)**	0.07 (0.01–0.44)**	0.10 (0.02–0.55)**	0.08 (0.01–0.46)**
Cigarette pack per day#	0.74 (0.34–1.64)	0.69 (0.32–1.48)	0.82 (0.42–1.59)	0.63 (0.32–1.27)
Low activity intensity	1.46 (0.22–9.61)	1.40 (0.24–8.25)	1.25 (0.22–7.22)	1.13 (0.21–6.05)
Medium activity intensity	0.46 (0.06–3.61)	0.49 (0.07–3.52)	0.46 (0.07–3.33)	0.39 (0.06–2.71)
High activity intensity	5.98 (0.38–93.3)	6.26 (0.44–89.6)	5.03 (0.38–67.4)	5.22 (0.38–71.9)
Using fertilizer to treat gardens	2.97 (0.81–10.9)*	2.44 (0.73–8.17)	2.97 (1.01–8.76)**	2.43 (0.72–8.23)
Living near industry/sewage treatment plant/farm	1.16 (0.41–3.30)	1.15 (0.40–3.28)	1.87 (0.69–5.11)	2.25 (0.69–7.27)
Occupational exposure to metal	4.76 (0.39–58.8)	2.04 (0.27–15.3)	0.69 (0.10–4.84)	0.78 (0.12–5.08)
Occupational exposure to pesticide	6.95 (1.23–39.1)**	2.64 (0.47–14.8)	2.66 (0.47–14.9)	0.88 (0.12–6.69)
Occupational exposure to dust/fibers/fumes or gas	0.47 (0.06–3.77)	1.54 (0.29–8.12)	1.94 (0.37–10.2)	4.44 (0.69–28.4)
Occupational exposure to radiation	1.25 (0.35–4.47)	1.73 (0.37–8.14)	1.73 (0.46–6.50)	1.96 (0.37–10.3)

*, p<0.1;

**, p<0.05; OR, odds ratio.

# Cigarette packs per day is a continuous variable.

doi:10.1371/journal.pone.0101186.t003

Higher educational attainment was associated with a lower odds of ALS. ALS was not associated with smoking or physical activity. This applied to both categorical (e.g., smoking status) and continuous (e.g., cigarette packs per day) indicators, and to analyses stratified by age or gender. One residential factor associated with ALS was exposure to fertilizer by treating private yards/gardens (for all participants: ORs (95% CI) = 2.97 (0.81–10.9) and 2.97 (1.01–8.76) for exposure windows 1 (n = 40/29, cases/controls) and 3 (n = 31/15, cases/controls), respectively; Table 3). While this relationship (treating private yards/gardens) was marginally significant in cases, significance was maintained for three of the exposure time conditions, in analyses for younger individuals (<60 years of age), and in males. The second residential factor associated with ALS was living near industry/sewage treatment plants/farms for individuals below 60 years of age (n = 18/12, cases/controls; OR (95% CI) = 4.56 (0.75–27.7); Table S2), and for females (n = 23/16, cases/controls; OR (95% CI) = 5.18 (0.98–27.3); Table S3) for exposure window 3. The sample size did not permit further stratification or examination of interactions, such as analysis of both younger and female subjects.

Occupational exposure to pesticides was associated with ALS (n = 38/19, cases/controls; OR (95% CI) = 6.95 (1.23–39.1)) for exposures occurring over the last 30 years but not in other time frames (Table 3). Pesticide exposure was also associated with ALS in age- and gender-stratified analyses, although significance decreased (p = 0.037–0.090; Table S2 and Table S3). Occupational exposure to metals was uncommon among study participants. The most frequent metal exposures were welding fumes (n = 11/8, cases/controls), lead (n = 9/6), nickel (n = 3/5), mercury (n = 4/2), and chromates (n = 1/3). Given these small numbers, we considered exposure to any heavy metal. No association was observed with this composite measure in the present study. Likewise, no association between exposure to dust/fibers/fumes or gas was found in the final models for all participants. An association between dust/fibers/fumes or gas exposure and ALS was found among males for continuous exposures over the past 30 years (OR (95% CI) = 15.6 (1.38–177); Table S3). Women had an increased odds of ALS with exposure to occupational radiation occurring 10 to 30 years earlier (OR (95% CI) = 67.7 (2.50–999); Table S3). This was not found in men or for other exposure windows.

## Discussion

### Als Risk Factors

#### Education

Our finding that educational attainment was associated with a lower odds of ALS is consistent with case-control studies in Boston [14] and Pennsylvania [20], and may represent an interaction with exposure since subjects with more education are more likely to work and live in environments with lower exposure. Interaction terms between these variables (education and exposure risk factors) did not attain statistical significance in model 1 (exposure in the last 30 years), possibly due to the limited sample size for this analysis.

#### Smoking

Although cigarette smoking and tobacco smoke exposure may increase the odds of ALS via inflammation, oxidative stress, and neurotoxicity induced by heavy metals or other chemicals present in cigarette smoke [21]–[24], we did not find an association between ALS and smoking. A large prospective study in the United States (414,493 male and 572,736 female participants; 617 ALS deaths among men and 539 among women) reported an increased risk of ALS with formaldehyde exposure, a component of cigarette smoke (women: RR = 1.67, 95% CI = 1.24–2.24, p = 0.002; men: RR = 0.69, 95% CI = 0.49–0.99, p = 0.04) [25]. A population-based case-control study in western Washington State (161 cases, 321 controls) showed an increased odds of ALS for ever-smokers (alcohol-adjusted OR = 2.0, 95% CI = 1.3–3.2), current smokers (OR = 1.5, 95% CI = 0.9–2.4), and for smoking duration and cigarette pack-years [26]. Conversely, negative results were obtained in a prospective study in the United Kingdom, although ever-smoking females had an increased risk of ALS (RR = 1.53, 95% CI = 1.04–2.23) [27]. Another prospective study in the United States (459,360 male and 638,849 female participants; 330 ALS deaths among men and 291 among women) associated cigarette smoking with increased ALS mortality in women, but not men (RR = 1.67, 95% CI = 1.24–2.24) [28]. Such inconsistencies likely arise from limitations related to sample design (most were case-control studies, not prospective cohort studies), small sample sizes, the use of hospital controls with a high frequency of smoking, interactions with other variables such as educational attainment, the use of spouses and/or friends as controls that may overmatch with respect to tobacco use, and other unaccounted for factors [29]. Nevertheless, in an updated literature review of 7 articles meeting the author's inclusion criteria, it was concluded that “smoking may be considered an established risk factor of sporadic ALS” [30].

#### Physical Activity

The reported disproportionate increase in ALS incidence among professional athletes, which has received considerable attention, has prompted many investigations into the potential role of physical activity in the development of ALS, which may result through increased risk of exposure to toxins, increased transport of the toxins, and increased susceptibility of target cells to injury from the toxins [31]. The present study did not find an association between ALS physical activity. Similarly, studies that have separated work and leisure-related physical activity have also been inconsistent [14],[32]. Accurate and quantitative assessment of past physical activity is challenging, as the number or type of activities reported in a survey may not reflect energy expenditures. Longstreth et al used a more sophisticated metabolic measure, metabolics equivalents for each activity weighted by the annual number of hours spent at each activity, but still found no association with ALS [32]. The present analysis did not include physical activity information prior to the five year recall period or direct measures of work-related physical activity. Several case reports have associated physical trauma and ALS development [33]. An older review identified heavy labor as a risk factor [12], and a case-control study among New England construction workers (109 cases, 253 controls) found elevated odds (OR = 2.9, 1.2–7.2) [15]. However, negative findings were reported in western Washington State (174 cases, 348 controls) [32] and in a prospective case-control study (95 cases, 106 controls) examining physical activity and trauma [34]. Like the environmental factors discussed earlier, evidence pertaining to the role of physical activity in ALS causation remains inconclusive.

#### Residential Factors And Chemicals

ALS has been associated with exposure to a number of chemicals, with most of the supporting evidence implicating agricultural chemicals such as pesticides, fertilizers, herbicides, and insecticides. Our study showed an association between an exposure to fertilizer and ALS. Information regarding residential exposure to fertilizers was obtained for only the current dwelling, which likely explains the stronger association for the last 10 years compared to the other time frames. It is also reasonable that younger males are more likely to perform yard and gardening work. Similar findings were reported in Australia for 179 case-control pairs [35]. Regular gardening (non-occupational exposure) was significantly associated with ALS (OR = 6.64, 95% CI = 1.61–27.4) for all subjects. After stratified by gender, the significant association only was shown in males (OR = 4.90, 95% CI = 1.11–21.7).

An association between a residence near industry and sewage treatment plants or farms for individuals less than 60 years of age was also demonstrated. Living near such facilities may involve exposure to a variety of air, water and soil pollutants. Possibly women in the cohort were less likely to be occupationally exposed to such pollutants and instead living near such facilities might provide exposures otherwise not encountered. Also, women may spend more time at home and thus experience greater exposure from nearby emission sources.

Pesticides initially aroused interest due to the increased risk of ALS observed among United States veterans exposed to pesticides [36],[37]. In addition, pesticides have been implicated in other neurodegenerative diseases like Parkinson's and Alzheimer's disease [38],[39]. Our study shows an association between pesticide exposure and ALS. Pesticide exposure has been associated with ALS in a multiple studies [11],[40]. The same association was seen in a Boston study using self-reported exposures [14], and an Australian study also using self-reported exposures of both industrial and residential use of herbicides and pesticides [35]. Recently, a study with 66 pairs of age-, race-, and gender-matched cases and controls found a very similar result to the present study. A significant association between occupational exposure to pesticides and ALS (OR = 6.50, 95% CI = 1.78–23.77) was shown after adjusting education, smoking, and other occupational exposures, including metals, solvents, and electromagnetic fields [20]. Pesticide exposure itself is associated with farming, living on or near farms, and the use of well water, and higher incidence rates of ALS have been found in United States rural farm areas west of the Mississippi River [41] and among farm workers and shepherds in Greece and Italy [42],[43]. A systematic review study concluded that the pesticide exposure and ALS association was significant [13].

#### Metals

The role of heavy metals, especially lead, in neurodegenerative diseases has received considerable attention. We did not see an association in this study. Lead may play several roles in the onset and progression of ALS [44], and like pesticides, lead exposure has been widespread. Lead exposure has been linked to ALS in many case-control studies. In New England (109 cases and 256 controls), elevated blood and bone lead levels were associated with increased odds of ALS (OR = 1.9, 95% CI = 1.4–2.6) for each µg dl^−1^ increase in lead, with OR = 3.6 (95% CI = 0.6–20.6) for each unit increase in log-transformed patella Pb and OR = 2.3 (95% CI = 0.4–14.5) for each unit increase in log-transformed tibia lead [9]. In United States veterans (184 cases and 194 controls), elevated blood Pb levels were associated with ALS (OR = 1.9, 95% CI = 1.3–2.7) [16]. As a third example, in Boston (95 cases and 106 controls), self-reported lead exposure was associated with ALS (p = 0.02) [14]. Overall, however, results have not been consistent and many studies show negative outcomes [45]–[47]. In addition, other metals, especially mercury and cadmium, have been studied, but again results have been inconsistent [48]–[50]. Malek et al [20] showed that a composite measure of heavy metal exposure (lead and mercury) was significantly associated with increased risk of ALS (OR = 3.65). While lead exposure has been associated with ALS [14],[16], associations and causal mechanisms have not been shown for mercury, cadmium or other metals. In a small Japanese study (21 cases, 36 controls), mercury and selenium levels in plasma and blood cells were significantly lower for ALS patients in the late stage than controls due to their disability, including consuming a liquid diet [51]. A very small study (9 cases) in Italy showed significantly higher cadmium blood levels in cases than in controls (after excluding advanced cases with the worst functional impairment) [50]. Grouping exposure to different metals together may result in exposure misclassification and diminished ability to detect associations, a limitation of the present as well as earlier studies [13].

#### Dust/fibers/fumes

Several studies have indirectly implicated occupational exposure to particulate matter with ALS [11],[40],[52]. We did not see an association in our study. Airborne dust, fumes, and fibers found in some occupational settings may represent an important exposure to airborne particulate matter. Exposure to particulate matter in ambient air, much of which arises from combustion sources, is widespread but concentrations are typically far below those in occupational settings. Particulate matter exposures have been examined with respect to neurological outcomes in a number of studies [53]–[55], and associated with ALS in several occupational settings [11],[40],[52]. The occupational settings investigated (veterinarians, hairdressers, graders and sorters (non-agricultural)), however, were likely to have elevated co-exposures of solvents, metals, and possibly other agents. No study has directly evaluated associations between environmental exposure of particulate matter and ALS.

#### Radiation/electromagnetic Fields

Radiation has been considered as a potential risk factor for ALS since a myeloradiculopathy presentation can be caused by electrical injuries with a long latency period [56]. We saw an association with ALS in women who had an exposure to occupational radiation 10 to 30 years earlier; however, the very large OR, the small number of exposure cases (n = 10/2 for cases/controls), and gender effect specificity suggest that results may not be meaningful. Three studies previously reported associations between radiation or electromagnetic field exposure [57]–[59]; thus, further investigation of such exposures is warranted. Radiation has been investigated in many case-control studies, again with inconsistent results. Electrical-related occupations (OR = 1.3, 95% CI = 1.1–1.6) [59], and exposure to electromagnetic fields (OR = 2.3, 95% CI = 1.29–4.09) [57] were associated with ALS. In a cohort mortality study at five large United States electric utility companies (139,905 men), mortality from ALS was associated with the duration of work in jobs with electromagnetic field exposure (RR = 2.0, 95% CI = 1.0–9.8) [60]. A prospective case-control study in Denmark of utility workers (n = 30,631, 81% male), however, did not find significant linkages between electromagnetic field exposure and neurological diseases [61].

### Study Limitations And Recommendations

Case-control studies assessing environmental risk factors in ALS are pursued due to low cost, efficiency, disease latency, and tendency to affect older individuals [16],[57]. While a few prospective cohort studies have been conducted [25],[34],[62], such designs are challenging given the often long period between exposure and ALS diagnosis [63]. Limitations of any case-control study, including our own, include small sample size, selection bias, environment exposure misclassification, recall bias, and confounding.

Typical sizes for ALS case-control studies are about 100 to 400 participants [13]. Larger studies benefit from greater power and enhanced ability to evaluate small effects and interactions. Thus, another limitation of this study is the relatively small sample size; however, our current goal is to recruit 600 participants which should address this concern.

While cases and controls were matched on both age and gender, several differences are worth mention. Recruiting patients via University of Michigan resources may attract more educated control compared to case volunteers. As educational attainment may be related to working and living in an environment with fewer exposures and risk factors, our data may have selection bias [64],[65].

Exposure misclassification is also a concern in epidemiology studies. Data was collected using self-reported questionnaires that queried an exhaustive range of chemicals and potential exposures, designed to aid in subject recall. Nonetheless, individuals may be unaware of exposures and potential risks. In addition, two important challenges in estimating exposure are the long latency and recall period and the large number of toxins of interest. This suggests that a blended approach that combines questionnaires, exposure modeling (e.g., using residence information to evaluate past air pollution exposure), biological measurements (e.g., bone or blood lead measurements), and possibly environmental monitoring data (e.g., water quality measurements) could provide greater specificity of exposures and accuracy.

Recall bias is also a limitation when using questionnaires, especially when seeking long term or historical information regarding exposure as these and other factors of possible significance may have occurred many years before ALS diagnosis and study enrollment, and thus participants may have difficulty remembering potential data. We used self-reported exposure, several time periods, and considered exposures up to 30 years earlier. Future studies are needed to confirm exposures using biomarkers.

Risk factors such as educational attainment and smoking status may act as confounders or effect modifiers that alter results of statistical models. As previously noted, controls were more likely to have a higher educational attainment, which may affect exposure. Including education variables in the regression models should help adjust for potential differences, but does not eliminate the potential for confounding; however, we obtained comparable results when analyses were stratified by educational attainment, suggesting this was not an issue (Table S1).

## Conclusions

This interim analysis of a larger ongoing case-control study shows an association of fertilizers and pesticides with an increased odds of developing ALS in a randomly selected ALS subjects compared to controls. These results were largely consistent over multiple time frames, as well as in analyses stratified by age and gender. Smoking, occupational exposures to metals, dust/fibers/fumes/gas and radiation, and physical activity were not associated with ALS. While consistent with earlier literature, these associations should be interpreted cautiously given the relatively modest sample size and other study limitations. The study is innovative in its use of different exposure periods and the wide range of exposures and covariates considered. Future studies could build on our methods by increasing sample size, using face-to-face interviews and trained interviewers (possibly with pictorial methods to increase awareness of exposures), obtaining exposure information using exposure models and biomonitoring, quantifying physical activity, and including information regarding income and alcohol consumption.

## Supporting Information

Table S1 Demographics, smoking and physical activities in leisure time, stratified by gender. (DOCX)

Table S2 Results of multiple regression models at four exposure windows stratified by age group. (DOCX)

Table S3 Results of multiple regression models at four exposure windows stratified by gender. (DOCX)
