# Supplementary material for: Environmental Risk Factors and Amyotrophic Lateral Sclerosis (ALS): A Case-Control Study of ALS in Michigan
Source: PLoS One. 2014 Jun 30;9(6):e101186. doi: 10.1371/journal.pone.0101186 (PMC4076303; doi:10.1371/journal.pone.0101186)
Supplement: Table S1 — (DOCX) [file pone.0101186.s001.docx]

#### Table S1. Demographics, smoking and physical activities in leisure time, stratified by gender.

| Variable | Group | Female | | |  | Male | | |
| --- | --- | --- | --- | --- | --- | --- | --- | --- |
|  |  | Cases=31 | Controls=31 | p-value |  | Cases=35 | Controls=35 | p-value |
|  |  | Number (%) | Number (%) |  |  | Number (%) | Number (%) |  |
| Age of consent | 40-49 | 1 (3.23) | 1 (3.23) | 1.000 |  | 7 (20.00) | 7 (20.00) | 1.000 |
|  | 50-59 | 6 (19.35) | 6 (19.35) |  |  | 11 (31.43) | 11 (31.43) |  |
|  | 60-69 | 16 (51.61) | 16 (51.61) |  |  | 11 (31.43) | 11 (31.43) |  |
|  | 70-79 | 8 (25.81) | 8 (25.81) |  |  | 6 (17.14) | 6 (17.14) |  |
|  | 80-89 | 0 (0.00) | 0 (0.00) |  |  | 0 (0.00) | 0 (0.00) |  |
| Education | ≤ High school | 12 (38.71) | 2 (6.45) | 0.002** |  | 10 (28.57) | 1 (2.86) | 0.003** |
|  | > High school | 19 (61.29) | 29 (93.55) |  |  | 25 (71.43) | 34 (97.14) |  |
| Marital status | Married | 18 (58.06) | 13 (41.94) | 0.338 |  | 27 (77.14) | 17 (48.57) | 0.079* |
|  | Widowed | 7 (22.58) | 4 (12.90) |  |  | 0 (0.00) | 1 (2.86) |  |
|  | Divorced | 2 (6.45) | 7 (22.58) |  |  | 6 (17.14) | 9 (25.71) |  |
|  | Separated | 1 (3.23) | 1 (3.23) |  |  | 0 (0.00) | 0 (0.00) |  |
|  | Never married | 2 (6.45) | 5 (16.13) |  |  | 1 (2.86) | 7 (20.00) |  |
|  | Living with partner | 1 (3.23) | 1 (3.23) |  |  | 1 (2.86) | 1 (2.86) |  |
|  | Married | 18 (58.06) | 13 (41.94) | 0.204 |  | 27 (77.14) | 17 (48.57) | 0.013** |
|  | Un-married | 13 (41.94) | 18 (58.06) |  |  | 8 (22.86) | 18 (51.43) |  |
| Smoking status | Never-smoker | 16 (51.61) | 16 (51.61) | 0.831 |  | 14 (40.00) | 13 (37.14) | 0.965 |
|  | Former-smoker | 13 (41.94) | 14 (45.16) |  |  | 14 (40.00) | 15 (42.86) |  |
|  | Current-smoker | 2 (6.45) | 1 (3.23) |  |  | 7 (20.00) | 7 (20.00) |  |
| Cigarette packs/day | Never | 16 (51.61) | 16 (51.61) | 0.524 |  | 14 (40.00) | 13 (37.14) | 0.881 |
|  | < 1 pack/day | 4 (12.90) | 7 (22.58) |  |  | 6 (17.14) | 5 (14.29) |  |
|  | ≥ 1 pack/day | 11 (35.48) | 8 (25.81) |  |  | 15 (42.86) | 17 (48.57) |  |
| Number of years of smoking | Never | 16 (51.61) | 16 (51.61) | 0.183 |  | 14 (40.00) | 13 (37.14) | 0.836 |
|  | < 20 years | 4 (12.90) | 9 (29.03) |  |  | 6 (17.14) | 8 (22.86) |  |
|  | ≥ 20 years | 11 (35.48) | 6 (19.35) |  |  | 15 (42.86) | 14 (40.00) |  |
| Cigarette pack-years | Never | 16 (51.61) | 16 (51.61) | 0.038* |  | 14 (40.00) | 13 (37.14) | 0.964 |
|  | < 20 pack-years | 4 (12.90) | 11 (35.48) |  |  | 8 (22.86) | 8 (22.86) |  |
|  | ≥ 20 pack-years | 11 (35.48) | 4 (12.90) |  |  | 13 (37.14) | 14 (40.00) |  |
| Physical activities | Jogging, running | 7 (22.58) | 4 (12.90) | 0.319 |  | 11 (31.43) | 11 (31.43) | 1.000 |
|  | Bicycling | 12 (38.71) | 16 (51.61) | 0.307 |  | 21 (60.00) | 17 (48.57) | 0.337 |
|  | Swimming | 4 (12.90) | 10 (32.26) | 0.068* |  | 14 (40.00) | 7 (20.00) | 0.068* |
|  | Aerobic dancing | 7 (22.58) | 7 (22.58) | 1.000 |  | 3 (8.57) | 2 (5.71) | 1.000 |
|  | Recreational dancing | 11 (35.48) | 4 (12.90) | 0.038** |  | 7 (20.00) | 4 (11.43) | 0.325 |
|  | Calisthenics | 11 (35.48) | 14 (45.16) | 0.437 |  | 13 (37.14) | 13 (37.14) | 1.000 |
|  | Gardening, yard work | 23 (74.19) | 23 (74.19) | 1.000 |  | 31 (88.57) | 23 (65.71) | 0.023** |
|  | Weightlifting | 6 (19.35) | 7 (22.58) | 0.755 |  | 11 (31.43) | 11 (31.43) | 1.000 |
|  | Soccer, football, baseball, field hockey, golf | 4 (12.90) | 5 (16.13) | 1.000 |  | 20 (57.14) | 12 (34.29) | 0.055* |
|  | Ice hockey, tennis, boxing, wresting | 0 (.00) | 1 (3.23) | 1.000 |  | 9 (25.71) | 4 (11.43) | 0.124 |
|  | Other | 8 (25.81) | 5 (16.13) | 0.3493 |  | 8 (22.86) | 11 (31.43) | 0.4201 |
| Physical activity intensity (excluding others) | Never | 4 (12.90) | 2 (6.45) | 0.461 |  | 1 (2.86) | 3 (8.57) | 0.286 |
|  | Low (0-3 activities) | 19 (61.29) | 18 (58.06) |  |  | 16 (45.71) | 18 (51.43) |  |
|  | Medium (4-6 activities) | 7 (22.58) | 11 (35.48) |  |  | 13 (37.14) | 13 (37.14) |  |
|  | Highs (7+ activities) | 1 (3.23) | 0 (0.00) |  |  | 5 (14.29) | 1 (2.86) |  |
| Physical activity intensity (including others) | Never | 4 (12.90) | 2 (6.45) | 0.661 |  | 1 (2.86) | 2 (5.71) | 0.054* |
|  | Low (0-3 activities) | 17 (54.84) | 15 (48.39) |  |  | 14 (40.00) | 17 (48.57) |  |
|  | Medium (4-6 activities) | 8 (25.81) | 12 (38.71) |  |  | 11 (31.43) | 15 (42.86) |  |
|  | Highs (7+ activities) | 2 (6.45) | 2 (6.45) |  |  | 9 (25.71) | 1 (2.86) |  |

*, p<0.1; **, p<0.05.
