# Supplementary material for: Environmental Risk Factors and Amyotrophic Lateral Sclerosis (ALS): A Case-Control Study of ALS in Michigan
Source: PLoS One. 2014 Jun 30;9(6):e101186. doi: 10.1371/journal.pone.0101186 (PMC4076303; doi:10.1371/journal.pone.0101186)
Supplement: Table S2 — (DOCX) [file pone.0101186.s002.docx]

#### Table S2. Results of multiple regression models at four exposure windows stratified by age group.

| Exposure Window | Risk Factors | Age < 60 years | | | Age ≥ 60 years | | |
| --- | --- | --- | --- | --- | --- | --- | --- |
|  |  | (n = 50) | | | (n = 82) | | |
|  |  | OR | 95% CI | | OR | 95% CI | |
| 1. Exposure in the last 30 years | Education ≥ high school | 0.01** | <0.001 | 0.41 | 0.13** | 0.02 | 0.72 |
|  | Cigarette pack per day# | 0.42 | 0.11 | 1.66 | 1.01 | 0.48 | 2.11 |
|  | Low activity intensity | 3.70 | 0.10 | 143.29 | 0.61 | 0.05 | 8.18 |
|  | Medium activity intensity | 3.75 | 0.10 | 136.56 | 0.27 | 0.02 | 3.68 |
|  | High activity intensity | 25.99 | 0.34 | >999 | 3.82 | 0.11 | 131.12 |
|  | Using fertilizer to treat gardens | 19.35** | 2.12 | 176.28 | 1.71 | 0.55 | 5.26 |
|  | Living near industry/sewage treatment plant/farm | 1.98 | 0.32 | 12.07 | 0.98 | 0.26 | 3.73 |
|  | Occupational exposure to metal | 1.94 | 0.11 | 34.23 | 0.86 | 0.07 | 9.98 |
|  | Occupational exposure to pesticide | 12.01* | 0.93 | 155.43 | 3.84* | 0.81 | 18.14 |
|  | Occupational exposure to dust/fibers/fumes or gas | 3.87 | 0.29 | 52.50 | 0.62 | 0.07 | 5.73 |
|  | Occupational exposure to radiation | 0.51 | 0.07 | 3.88 | 1.41 | 0.30 | 6.68 |
| 2. Exposure in the last 10 years | Education ≥ high school | 0.01** | <0.001 | 0.60 | 0.12** | 0.02 | 0.64 |
|  | Cigarette pack per day# | 0.17* | 0.03 | 1.11 | 1.00 | 0.47 | 2.15 |
|  | Low activity intensity | 16.96 | 0.27 | >999 | 0.51 | 0.04 | 6.98 |
|  | Medium activity intensity | 19.70 | 0.21 | >999 | 0.27 | 0.02 | 3.75 |
|  | High activity intensity | 146.16* | 0.98 | >999 | 2.94 | 0.09 | 97.24 |
|  | Using fertilizer to treat gardens | 16.04** | 1.44 | 179.06 | 1.53 | 0.49 | 4.79 |
|  | Living near industry/sewage treatment plant/farm | 1.39 | 0.22 | 8.74 | 0.71 | 0.20 | 2.47 |
|  | Occupational exposure to metal | 1.58 | 0.10 | 24.66 | 1.15 | 0.13 | 10.10 |
|  | Occupational exposure to pesticide | 35.24** | 1.25 | 997.00 | 2.20 | 0.21 | 22.75 |
|  | Occupational exposure to dust/fibers/fumes or gas | 10.77 | 0.61 | 189.45 | 0.86 | 0.08 | 9.66 |
|  | Occupational exposure to radiation | 0.58 | 0.05 | 6.64 | 1.29 | 0.22 | 7.40 |
| 3. Exposure in the period from 30 years ago to 10 years ago | Education ≥ high school | 0.02** | <0.001 | 0.65 | 0.14** | 0.02 | 0.88 |
|  | Cigarette pack per day# | 1.16 | 0.32 | 4.27 | 0.97 | 0.47 | 1.98 |
|  | Low activity intensity | 0.70 | 0.02 | 21.05 | 0.94 | 0.07 | 12.33 |
|  | Medium activity intensity | 0.32 | 0.01 | 14.39 | 0.34 | 0.03 | 4.49 |
|  | High activity intensity | 9.56 | 0.19 | 484.56 | 7.13 | 0.20 | 249.78 |
|  | Using fertilizer to treat gardens | 60.56** | 3.80 | 966.22 | 2.53 | 0.76 | 8.39 |
|  | Living near industry/sewage treatment plant/farm | 4.56* | 0.75 | 27.65 | 1.98 | 0.57 | 6.84 |
|  | Occupational exposure to metal | 0.83 | 0.07 | 9.74 | 0.30 | 0.04 | 2.33 |
|  | Occupational exposure to pesticide | 1.66 | 0.12 | 23.53 | 5.41* | 0.90 | 32.47 |
|  | Occupational exposure to dust/fibers/fumes or gas | 6.24 | 0.56 | 69.05 | 0.84 | 0.15 | 4.72 |
|  | Occupational exposure to radiation | 0.56 | 0.08 | 3.92 | 3.67 | 0.56 | 24.19 |
| 4. Continuous Exposure in the last 30 years | Education ≥ high school | 0.07* | 0.00 | 1.26 | 0.11** | 0.02 | 0.64 |
|  | Cigarette pack per day# | 0.67 | 0.18 | 2.47 | 0.90 | 0.45 | 1.82 |
|  | Low activity intensity | 3.96 | 0.12 | 126.64 | 0.70 | 0.05 | 9.01 |
|  | Medium activity intensity | 2.04 | 0.04 | 113.73 | 0.34 | 0.03 | 4.51 |
|  | High activity intensity | 23.30 | 0.40 | >999 | 4.34 | 0.14 | 139.64 |
|  | Using fertilizer to treat gardens | 10.67** | 1.17 | 97.50 | 2.00 | 0.64 | 6.27 |
|  | Living near industry/sewage treatment plant/farm | 2.06 | 0.44 | 9.69 | 1.04 | 0.34 | 3.18 |
|  | Occupational exposure to metal | 0.82 | 0.09 | 7.29 | 0.42 | 0.06 | 3.12 |
|  | Occupational exposure to pesticide | 3.41 | 0.22 | 52.42 | 1.77 | 0.18 | 17.42 |
|  | Occupational exposure to dust/fibers/fumes or gas | 5.73 | 0.68 | 48.31 | 1.39 | 0.19 | 10.09 |
|  | Occupational exposure to radiation | 0.73 | 0.10 | 5.45 | 3.88 | 0.37 | 40.39 |

#### *, p<0.1; **, p <0.05; OR, odds ratio.

# Cigarette packs per day is a continuous variable.
