# Supplementary material for: Environmental Risk Factors and Amyotrophic Lateral Sclerosis (ALS): A Case-Control Study of ALS in Michigan
Source: PLoS One. 2014 Jun 30;9(6):e101186. doi: 10.1371/journal.pone.0101186 (PMC4076303; doi:10.1371/journal.pone.0101186)
Supplement: Table S3 — (DOCX) [file pone.0101186.s003.docx]

#### Table S3. Results of multiple regression models at four exposure windows stratified by gender.

| Exposure Window | Risk Factors | Female | | | Male | | |
| --- | --- | --- | --- | --- | --- | --- | --- |
|  |  | (n = 62) | | | (n = 70) | | |
|  |  | OR | 95% CI | | OR | 95% CI | |
| 1. Exposure in the last 30 years | Education ≥ high school | 0.16* | 0.02 | 1.06 | 0.03** | 0.00 | 0.35 |
|  | Cigarette pack per day# | 1.06 | 0.33 | 3.47 | 0.97 | 0.41 | 2.25 |
|  | Low activity intensity | 1.00 | 0.09 | 11.30 | 0.59 | 0.03 | 13.97 |
|  | Medium activity intensity | 0.24 | 0.01 | 4.04 | 0.54 | 0.02 | 12.62 |
|  | High activity intensity | 1.91 | 0.05 | 77.35 | 11.97 | 0.25 | 577.56 |
|  | Using fertilizer to treat gardens | 2.30 | 0.61 | 8.65 | 4.58* | 0.98 | 21.30 |
|  | Living near industry/sewage treatment plant/farm | 1.95 | 0.39 | 9.79 | 0.52 | 0.12 | 2.33 |
|  | Occupational exposure to metal | 0.34 | 0.01 | 14.86 | 1.23 | 0.11 | 13.68 |
|  | Occupational exposure to pesticide | 4.13 | 0.48 | 35.22 | 5.17* | 0.88 | 30.48 |
|  | Occupational exposure to dust/fibers/fumes or gas | 0.85 | 0.07 | 10.48 | 2.19 | 0.23 | 20.94 |
|  | Occupational exposure to radiation | 5.36* | 0.83 | 34.57 | 0.46 | 0.08 | 2.51 |
| 2. Exposure in the last 10 years | Education ≥ high school | 0.20* | 0.03 | 1.20 | 0.03** | 0.00 | 0.39 |
|  | Cigarette pack per day# | 1.32 | 0.43 | 4.10 | 0.78 | 0.33 | 1.82 |
|  | Low activity intensity | 1.06 | 0.09 | 12.18 | 0.73 | 0.03 | 16.40 |
|  | Medium activity intensity | 0.40 | 0.03 | 6.20 | 0.86 | 0.04 | 19.92 |
|  | High activity intensity | 1.61 | 0.06 | 45.60 | 11.85 | 0.23 | 602.96 |
|  | Using fertilizer to treat gardens | 2.77 | 0.74 | 10.36 | 3.18 | 0.69 | 14.71 |
|  | Living near industry/sewage treatment plant/farm | 1.16 | 0.28 | 4.83 | 0.42 | 0.10 | 1.74 |
|  | Occupational exposure to metal | 1.99 | 0.05 | 79.07 | 1.04 | 0.15 | 7.12 |
|  | Occupational exposure to pesticide | 2.78 | 0.30 | 25.63 | 2.12 | 0.22 | 20.04 |
|  | Occupational exposure to dust/fibers/fumes or gas | 0.64 | 0.05 | 9.02 | 3.44 | 0.43 | 27.81 |
|  | Occupational exposure to radiation | 1.32 | 0.20 | 9.01 | 1.80 | 0.26 | 12.48 |
| 3. Exposure in the period from 30 years ago to 10 years ago | Education ≥ high school | 0.26 | 0.03 | 2.04 | 0.03** | 0.00 | 0.36 |
|  | Cigarette pack per day# | 1.69 | 0.51 | 5.61 | 0.90 | 0.39 | 2.06 |
|  | Low activity intensity | 2.13 | 0.12 | 36.65 | 0.88 | 0.04 | 19.95 |
|  | Medium activity intensity | 0.22 | 0.01 | 4.83 | 0.40 | 0.02 | 9.35 |
|  | High activity intensity | 4.90 | 0.07 | 336.17 | 34.73* | 0.69 | >999 |
|  | Using fertilizer to treat gardens | 2.04 | 0.35 | 11.99 | 14.42** | 2.57 | 81.02 |
|  | Living near industry/sewage treatment plant/farm | 5.18* | 0.98 | 27.34 | 1.85 | 0.40 | 8.57 |
|  | Occupational exposure to metal | 0.08 | 0.00 | 4.45 | 0.41 | 0.04 | 3.77 |
|  | Occupational exposure to pesticide | 43.55* | 0.78 | >999 | 2.77 | 0.46 | 16.88 |
|  | Occupational exposure to dust/fibers/fumes or gas | 1.20 | 0.14 | 10.40 | 4.97 | 0.62 | 39.70 |
|  | Occupational exposure to radiation | 67.65** | 2.50 | >999 | 0.54 | 0.08 | 3.50 |
| 4. Continuous Exposure in the last 30 years | Education ≥ high school | 0.32 | 0.05 | 2.33 | 0.02** | 0.00 | 0.34 |
|  | Cigarette pack per day# | 1.79 | 0.58 | 5.56 | 0.72 | 0.31 | 1.66 |
|  | Low activity intensity | 1.43 | 0.10 | 19.96 | 0.73 | 0.03 | 17.94 |
|  | Medium activity intensity | 0.31 | 0.02 | 5.53 | 0.42 | 0.02 | 12.10 |
|  | High activity intensity | 2.00 | 0.06 | 72.36 | 28.30 | 0.40 | >999 |
|  | Using fertilizer to treat gardens | 1.63 | 0.34 | 7.79 | 17.09** | 2.54 | 114.95 |
|  | Living near industry/sewage treatment plant/farm | 3.12 | 0.71 | 13.70 | 1.13 | 0.26 | 4.99 |
|  | Occupational exposure to metal | 1.31 | 0.07 | 24.88 | 0.29 | 0.04 | 2.45 |
|  | Occupational exposure to pesticide | 4.96 | 0.31 | 79.78 | 0.87 | 0.07 | 11.61 |
|  | Occupational exposure to dust/fibers/fumes or gas | 0.94 | 0.12 | 7.41 | 15.60** | 1.38 | 176.66 |
|  | Occupational exposure to radiation | 8.11 | 0.36 | 180.54 | 1.85 | 0.22 | 15.29 |

#### *, p<0.1; **, p <0.05; OR, odds ratio.

# Cigarette packs per day is a continuous variable.
